# Comparative mitogenomic analyses of three North American stygobiont amphipods of the genus *Stygobromus* (Crustacea: Amphipoda)

**DOI:** 10.1080/23802359.2016.1174086

**Published:** 2016-11-21

**Authors:** Aaron William Aunins, David L. Nelms, Christopher S. Hobson, Timothy L. King

**Affiliations:** a Leetown Science Center, Aquatic Ecology Branch, Natural Systems Analysts, Inc, Kearneysville, WV, USA;; b United States Geological Survey, Virginia Water Science Center, Richmond, VA, USA;; c Virginia Natural Heritage Program, Richmond, VA, USA;; d United States Geological Survey, Leetown Science Center, Aquatic Ecology Branch, Kearneysville, WV, USA

**Keywords:** Amphipoda, mitogenomics, *Stygobromus indentatus*, *Stygobromus foliatus*, *Stygobromus tenuis potomacus*

## Abstract

The mitochondrial genomes of three North American stygobiont amphipods *Stygobromus tenuis potomacus*, *S. foliatus* and *S. indentatus* collected from Caroline County, VA, were sequenced using a shotgun sequencing approach on an Illumina NextSeq500 (Illumina Inc., San Diego, CA). All three mitogenomes displayed 13 protein-coding genes, 22 tRNAs and two rRNAs typical of metazoans. While *S. tenuis* and *S. indentatus* displayed identical gene orders similar to the pancrustacean ground pattern, *S. foliatus* displayed a transposition of the *trnL2*-*cox2* genes to after *atp8-atp6*. In addition, a short *atp8* gene, longer *rrnL* gene and large inverted repeat within the Control Region distinguished *S. foliatus* from *S. tenuis potomacus* and *S. indentatus*. Overall, it appears that gene order varies considerably among amphipods, and the addition of these *Stygobromus* mitogenomes to the existing sequenced amphipod mitogenomes will prove useful for characterizing evolutionary relationships among various amphipod taxa, as well as investigations of the evolutionary dynamics of the mitogenome in general.

## Introduction

The mid-Atlantic United States harbours a diverse array of stygobiont fauna associated with shallow groundwater habitats with over 14 species of amphipods within the genus *Stygobromus* described from within this region (Holsinger et al. 2011; Culver et al. 2012). Because these species occupy shallow groundwater habitats, they are particularly vulnerable to habitat degradation, and some species are threatened with extirpation.

Numerous amphipod mitogenomes have been sequenced recently revealing differences amongst distantly related species (Krebes & Bastrop 2012). Less common are mitogenomic comparisons among amphipods of the same genus. Pons et al. (2014) found that gene order was mostly conserved among seventeen amphipod species of the genus *Metacrangonyx*, whereas Stokkan et al. (2015) found a high level of rearrangement of protein coding genes among three amphipod species of the genus *Pseudoniphargus*. To provide additional insight into amphipod mitogenomic structure and evolution, we sequenced the mitogenomes of three species of amphipods in the genus *Stygobromus* to include *Stygobromus tenuis potomacus*, *S. indentatus* and *S. foliatus* and performed a brief comparative analysis with other amphipod mitogenomes.

## Methods

*Stygobromus tenuis potomacus* (38.1691 °N–77.2781°W), *S. indentatus* (38.0987 °N–77.1476 °W) and *S. foliatus* (38.1995 °N–77.2905 °W) were collected by hand from within leaf litter at seepage springs at Fort A.P. Hill, Caroline County, VA, in March 2014, and field preserved in 95% ethanol. Species identification was performed using taxonomic keys and published species descriptions presented in Holsinger (1967, 1969, 1978) and Holsinger et al. (2011). Specimens are stored at the US Geological Survey Leetown Science Center, Kearneysville, WV. Libraries were prepared from whole genomic DNA using the TruSeq Nano library preparation kit (Illumina, San Diego, CA) for 2 × 150bp paired-end sequencing on an Ilumina NextSeq500 sequencer (Illumina, San Diego, CA).

The annotated mitochondrial genomes of 26 amphipods were used for comparative analyses (Table 1). The location of protein coding genes was determined using the online program DOGMA (Wyman et al. 2004). Annotation of start and stop codons was based on extensive comparisons of candidate protein coding genes to the annotation of the same genes in other amphipod mitogenomes. Annotation of the large ribosomal subunit (rrnL) was defined as all base pairs between the adjacent tRNAs. In contrast, the ends of the small ribosomal subunits (rrnS) were determined through alignment with other amphipod *rrnS* genes to look for conserved regions indicating the likely start of *rrnS*, as well as the end of *rrnS* and the start of the control region. The location and secondary structures of the tRNA genes were identified through the automated online mitogenome annotation software MITOS (Bernt et al. 2013). A maximum likelihood phylogenetic tree with 500 bootstrap replications was created in the program MEGA7 (Kumar et al. 2016) using a MUSCLE alignment (Edgar 2004) generated in the program TranslatorX (Abascal et al. 2010) of thirteen protein coding genes from 23 other selected members of the order Amphipoda.

**Table 1.** Species names, GenBank accession numbers and mitogenome length of 26 amphipod mitogenomes used for comparative analyses with *Stygobromus tenuis*, *S. foliatus* and *S. indentatus*.

Species name	GenBank Accession	Mitogenome length	Complete mitogenome?
*Metacrangonyx remyi*	HE860512	14,787	No
*Metacrangonyx boveei*	HE860498	15,012	Yes
*Metacrangonyx ilvanus*	HE860503	14,770	No
*Metacrangonyx repens*	HE860495	14,354	Yes
*Metacrangonyx dominicanus*	HE860499	14,536	No
*Metacrangonyx samanensis*	HE860505	14,057	No
*Metacrangonyx longipes*	HE861923	14,113	Yes
*Metacrangonyx goulmimensis*	HE860500	14,503	No
*Metacrangonyx nicoleae tamri*	HE860504	14,644	Yes
*Metacrangonyx spinicaudatus*	HE860506	15,037	Yes
*Metacrangonyx paurosexualis*	HE860507	12,542	No
*Longipodacrangonyx stocki*	HE860496	12,924	No
*Metacrangonyx boutini boutini*	HE860497	13,301	No
*Metacrangonyx longicaudus*	HE860509	14,710	No
*Metacrangonyx panousei*	HE860510	14,478	No
*Metacrangonyx notenboomi*	HE860513	14,277	No
*Bahadzia jaraguensis*	NC_019661	14,657	Yes
*Caprella mutica*	NC_014492	15,427	Yes
*Eulimnogammarus verrucosus*	NC_023104	15,315	Yes
*Gammarus duebeni*	NC_017760	15,651	Yes
*Gondogeneia antarctica*	NC_16192	18,424	Yes
*Onisimus nanseni*	NC_013819	14,734	Yes
*Pseudoniphargus daviui*	NC_019662	15,157	Yes
*Pseudoniphargus sorbasiensis*	LN871175	15,460	No
*Pseudoniphargus gorbeanus*	LN871176	14,178	No
*Parhyale hawaiensis*	AY639937	12,224	No

## Results and discussion

**Figure 1.**
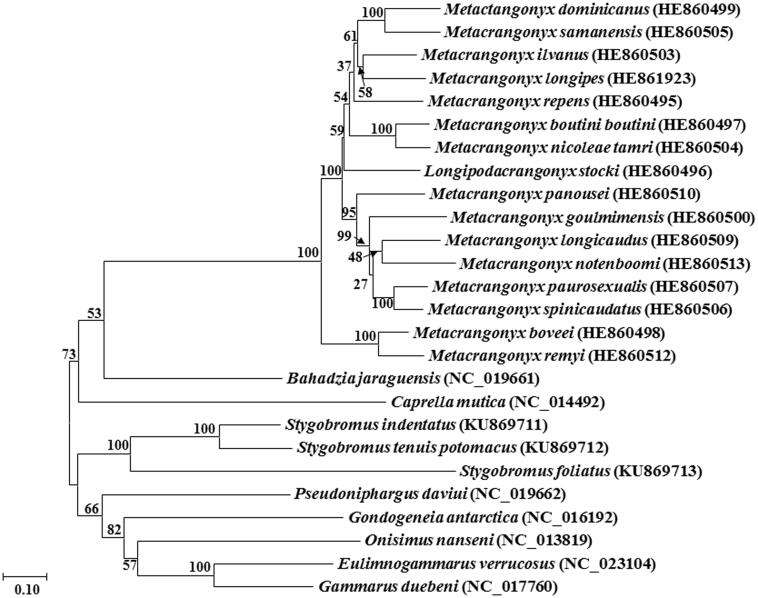
Phylogenetic tree indicating the relationships of *Stygobromus tenuis potomacus*, *S. indentatus* and *S. foliatus* with 23 other members of the order Amphipoda generated using the maximum-likelihood method based on 13 complete protein-coding genes from the mitochondrial genome. GenBank accession numbers are in parentheses next to the species names.

The complete mitogenomes of *S. tenuis potomacus* (14,915 bp, GenBank accession no. KU869712) and *S. indentatus* (14,638 bp, GenBank accession no. KU869711) were obtained along with the nearly complete mitogenome of *S. foliatus* (GenBank accession no. KU869713). The mitogenome of *S. foliatus* contained an inverted repeat of the control region, of which 365 bp were sequenced in each direction (bps 13,225–14,075) with the un-sequenced remainder annotated as a gap of unknown length. All mitogenomes consisted of 13 protein-coding genes, an *rrnS* and *rrnL* gene, and 22 tRNA genes typical of metazoans. In *S. tenuis potomacus* and *S. indentatus*, 23 genes were encoded on the + strand and 14 on the – strand, whereas *S. foliatus* had 22 genes on the + and 15 on the – strand. The same four protein-coding genes (*nad5*, *nad4*, *nad4l* and *nad1*) were on the – strand in all three species, which is a feature of the pancrustacean ground pattern (Boore et al. 1998). The AT% of *S. tenuis potomacus* and *S. indentatus* was 69%, which is typical of values observed in other amphipods. Results of the phylogenetic analysis (Figure 1) suggest that *S. tenuis potomacus*, *S. indentatus* and *S. foliatus* form a distinct well-supported clade.

*Stygobromus foliatus* had substantially more intergenic spacers (*n* = 23), which were often between protein coding genes, than was observed in *S. tenuis potomacus* (*n* = 9) or *S. indentatus* (*n* = 7) or any other examined amphipod mitogenome. In *S. foliatus*, we hypothesize these numerous intergenic spacers are a consequence of numerous transpositions and reverse transpositions of tRNAs.

Both *S. tenuis potomacus* and *S. indentatus* had identical gene orders, which were most similar to the pancrustacean ground pattern with the exception of transposition of some tRNAs (Boore et al. 1998; Pons et al. 2014), while *S. foliatus* displayed some unique rearrangements. *Stygobromus tenuis potomacus* and *S. indentatus* share the conserved gene block order of *cox1*–*trnL2*–*cox2* with other examined amphipods. In contrast, *S. foliatus* displays a transposition of *trnL2*–*cox2* downstream after *atp8*–*atp6*. *Stygobromus foliatus* also departed from the pancrustacean gene order of *nad5*–*trnH*–*nad4*–*nad4l*, with transposition of the *trnH* downstream between *nad2* and *trnG*. In addition, *S. foliatus* exhibited numerous reverse transposed tRNAs compared with the pancrustacean ground pattern and other examined amphipods.

Most protein-coding genes utilized ATN start codons, with ATG and ATA being the most frequent. Unconventional start codons include a TTG for *nad1* in *S. tenuis potomacus*, and a GTG start codon for the *nad4* gene in *S. foliatus,* which have not been used for these genes in other compared amphipod mitogenomes. Stop codons were most frequently TAA or TAG, although several genes ended with an incomplete T– – or TA–. These abbreviated stop codons are common in amphipods (Pons et al. 2014), and it is assumed that these are formed into complete terminal codons through post-transcriptional polyadenylation (Ojala et al. 1981).

Numerous deviations in length and overlap at some protein coding genes within *S. tenuis potomacus*, *S. indentatus*, and *S. foliatus* were observed. The *cox1* gene in *S. foliatus* was 20 bp longer than within *S. tenuis potomacus* and *S. indentatus*, with extra nucleotides near the start and the end of the gene accounting for the length difference. In all three species, the *cox1* gene began with two consecutive ATT-ATV start codons, which did not overlap with the adjacent tRNA, and which we annotated the first as the beginning of *cox1*. Several species of the genus *Metacrangonyx* have an extra candidate start codon for *cox1* overlapping the adjacent tRNA by 1 bp, but are annotated as beginning with the second start codon. Similarly other species such as *Onisimus nanseni* and *Gondogeneia antarctica* have alternate candidate start ATC start codons between the adjacent tRNA and their existing annotated start. The *atp8* gene within *S. foliatus* (105 bp) was substantially shorter than within *S. tenuis potomacus* (186 bp) and *S. indentatus* (177 bp), as well as in comparison with *atp8* within the other compared amphipod mitogenomes (range =156–162 bp). While no other described amphipods to date have such a short *atp8* gene as *S. foliatus*, Zhang et al (2013) described two species of rice planthoppers (subphylum Hexapoda) that possessed conspicuously short *atp8* genes of 99 and 102 bp, but the significance of a shortened *atp8* gene in any of these species is unknown. Another feature of the *S. foliatus atp8* gene was that it only overlapped with the adjoining *atp6* by one bp, whereas *S. tenuis* and *S. indentatus* had overlaps of 41 bp with *atp6*. These large overlaps between *atp6* and *atp8* have not yet been reported to our knowledge for any amphipod.

The *S. tenuis potomacus*, *S. foliatus* and *S. indentatus* mitogenomes all encoded 22 tRNA genes typical of metazoan mitochondrial genomes (Wolstenholme 1992). *Stygobromus tenuis potomacus* and *S. indentatus* possessed the same 14 tRNAs on the heavy strand, and eight tRNAs on the light strand. *Stygobromus foliatus* had 13 tRNAs on the heavy strand, and nine on the light strand, of which five were on different strands than within *S. tenuis potomacus* and *S. indentatus*. The lengths of the tRNAs ranged from 50 to 66 bps, and all formed typical cloverleaf-like secondary structures, though both *trnS1* and *trnS2* lacked the DHU arm, and *trnQ* lacked the TΨC stem.

The *rrnL* gene within *S. foliatus* when aligned with other amphipods possessed large overhangs on either end, and was longer than within *S. tenuis potomacus*, and *S. indentatus*. In the amphipod mitogenomes used for comparison in this study as well as in *S. tenuis potomacus* and *S. indentatus*, *rrnL* is flanked by *trnL1* and *trnV*, whereas in *S. foliatus* it is flanked by *trnS* and *trnQ*. Because the 3′ and 5′ ends of the *rrnL* gene are not well conserved, we have tentatively retained these overhangs in the annotation of *rrnL* in *S. foliatus*. In contrast to *S. foliatus*, the *S. indentatus* and *S. tenuis potomacus rrnL* genes were similar in length to *rrnL* within other amphipods.

An alignment of the *rrnS* gene among *S. foliatus*, *S. indentatus*, *S. tenuis potomacus* and other amphipod mitogenomes revealed a relatively conserved 5′ end. This resulted in annotation of a 46 bp spacer between the adjacent *trnI* and start of the *rrnS* gene in *S. foliatus*. On the 3′ end of the *rrnS* gene, *S. tenuis potomacus* and *S. indentatus* were annotated as ending at the start of a string of several T’s as was done for annotations of *rrnS* in *Gammarus duebeni* (Krebes & Bastrop 2012) and various species of Metacrangonyx (Pons et al. 2014). This string of T’s is interpreted as the beginning of the Control Region. In contrast, the *rrnS* gene in *S. foliatus* did not terminate at a string of T’s, but at an inversion of the control region. 365 bp were sequenced from both ends of the inversion, which was nearly perfect with greater than 99% sequence similarity when the ends were aligned. Large inverted repeats of the control region are characteristic of some amphipods as noted by Pons et al. (2014).

## Conclusions

Here, we have presented the complete mitogenomes of the stygobiont amphipods *S. tenuis potomacus* and *S. indentatus*, along with the nearly complete mitogenome of *S. foliatus*, adding to the growing number of available amphipod mitogenomes. While all three mitogenomes possessed the typical suite of genes found in other amphipods, some unique gene arrangements such as the transposition of *trnL2*–*cox2* downstream after *atp8*–*atp6* in *S. foliatus* has not been observed yet in any other amphipod, and differs from the pancrustacean ground pattern. While these rearrangements of protein-coding genes at the genus level are rare, they have been observed in other amphipod genera (Stokkan et al. 2015). Overall, these additional mitogenomes will prove useful for investigating phylogenetic relationships among various amphipod groups, and contribute to studies of mitogenome evolution in general.
